# Validation of the Spanish-language version of the Postpartum Specific Anxiety Scale Research Short Form: PSAS-ES-RSF

**DOI:** 10.1007/s00737-026-01695-1

**Published:** 2026-03-30

**Authors:** Natalia Costas-Ramón, Agustina María Vinagre-González, Sergio A. Silverio, Victoria Fallon, Paul Christiansen, Marta Evelia Aparicio-García

**Affiliations:** 1 Facultad de Ciencias de la Salud, University of Zaragoza, Zaragoza, Spain; 2 Facultad de Psicología, Complutense University of Madrid, Madrid, Spain; 3 Facultad de Derecho, Universidad Internacional De La Rioja, Logroño, Spain; 4 Department of Psychology, University of Liverpool, Liverpool, United Kingdom; 5 Department of Women & Children’s Health, King’s College London, London, United Kingdom; 6 Facultad de Psicología, Complutense University of Madrid, Madrid, Spain

**Keywords:** Postpartum anxiety, Postpartum Specific Anxiety Scale (PSAS), Maternal mental health, Psychometric validation, Postnatal emotional disorders, Maternal self-efficacy, Infant care anxieties, Psychosocial adjustment postpartum

## Abstract

**Background:**

Postpartum anxiety is a prevalent emotional disorder affecting approximately 20% of women, often more common than postpartum depression. Despite its high prevalence, the Spanish healthcare system lacks screening programs specifically targeting this condition.

**Methods:**

This study aimed to develop and validate a short version of the Postpartum Specific Anxiety Scale (PSAS-ES-RSF) to address time constraints in clinical practice and improve its usability as a screening tool. Using confirmatory factor analysis (CFA) on two independent Spanish samples (Sample 1: N = 699; Sample 2: N = 293), a 16-item version was extracted from the original 51-item PSAS-ES.

**Results:**

The four-factor structure of the PSAS-ES-RSF—addressing maternal competence, infant safety, practical infant care, and psychosocial adjustment—demonstrated strong psychometric properties (CFI > .95, RMSEA < .08). Cultural context and risk factors, such as maternal self-efficacy and the return to work, influenced the selection of items, showing some variation from the English-language short form.

**Conclusions:**

The PSAS-ES-RSF offers a reliable and efficient tool for exploring postpartum anxiety in Spanish-speaking populations. Further research is needed to explore its applicability in diverse cultural contexts and to continue refining postpartum anxiety screening strategies.

**Supplementary Information:**

The online version contains supplementary material available at 10.1007/s00737-026-01695-1.

## Background

### Postpartum anxiety

The postpartum period is a stage particularly prone to the development of emotional disorders (Epifanio et al. 2015; García-Esteve and Valdés Miyar 2017). These disorders occur in one out of every four women, with depression (approximately 15%), somatization (21.8%), and anxiety (23%) being the most common (Rostami et al. 2023; Azimi et al. 2023). Postpartum anxiety has a higher rate than more well studied conditions such as postpartum depression. Globally, signs of anxiety can be found in 20% of women during the postpartum period (Fairbrother et al. 2024; Gumilang and Linasari 2024). Despite the high prevalence of postpartum anxiety, the Spanish healthcare system lacks screening programmes and strategic care plans for both the prenatal and postnatal stages (Castejón and Sanz 2021; Olza et al. 2021).

The presence of pre-pregnancy emotional disorders, as well as poor general health of the mother, predisposes to the development of this condition (Field 2017; Zee-van et al. 2021). Risk factors related to parenting and birth include premature birth, a negative birth experience, excessive crying of the baby during the first week postpartum, and low perceived maternal self-efficacy (Zee-van et al. 2021). Maternal age (young mothers), higher educational levels, and being employed are variables associated with anxiety (Field 2017; Zee-van et al. 2021). Previous studies also highlight the importance of the mother having an adequate social and family support network postpartum. Lack of family support, marital/family conflicts, social health problems (Field 2017), and low partner support (Zee-van et al. 2021) are risk factors.

Postpartum anxiety impacts both the mother’s health and the health of the newborn, being a predictor of developmental problems in babies (Leonetti and Martins 2007). Effects include emotional problems (Navarrete et al. 2012), behavioral issues (Davies et al. 2021a), temperament (Field 2017), and impacts on the child’s cognitive and social development (Field 2017; Navarrete et al. 2012). It has demonstrated associations with sleep, health, internalizing behaviors, and conduct disorders in adolescents (Field 2017). The affective bond (Davies et al. 2021a), attachment (Bonacquisti et al. 2020), and mother–child interactions (Field 2017) are affected by this emotional disorder. Postpartum anxiety has negative effects on breastfeeding, leading to a lower likelihood of exclusive breastfeeding and a higher likelihood of early cessation (Fallon et al. 2016a; Davies et al. 2021b; Ionio et al. 2024).

### PSAS: a specific tool for postpartum anxiety

The World Health Organization emphasizes the need to include postpartum anxiety and depression screening in strategic health plans. It recommends the use of validated tools accompanied by referral and intervention circuits for women who need them (World Health Organization 2022).

Compared to other emotional disorders, postpartum anxiety is more challenging to explore, detect, and treat (Zappas et al. 2021) due, among others, to the difficulty in distinguishing physiological emotions (Misri et al. 2015) since some anxieties after birth are adaptive and allow adequate care. The lack of specific tools to differentiate physiological concerns and fears from an anxiety diagnosis is a significant barrier for professionals (Misri et al. 2015). Additionally, the stigmatization of emotional disorders in society and the perfectionism that some mothers impose on themselves in their maternal role can delay or prevent seeking professional help for fear of being judged (Mendoza and Saldivia 2015).

The Postpartum Specific Anxiety Scale (PSAS) was developed as an exploratory questionnaire for anxiety in the puerperium. The original English-language PSAS has proven to be a tool with excellent reliability and validity for researching this emotional disorder (Fallon et al. 2016b). Subsequent studies have reassessed its properties and studied the different tools available for detecting postpartum anxiety support and recommend its use as an exploratory resource for professionals (Gumilang and Linasari 2024; O’Carroll et al 2023).

The PSAS with four-factor structure has been translated into several languages and validated in different cultural contexts, demonstrating that it maintains its psychometric properties. The PSAS has translations and validations in French (Infante-Gil et al. 2021), Persian (Hasanzadeh et al. 2021), Chinese (Xu et al. 2021), and Italian (Ionio et al. 2023).

### Need for developing the short version: PSAS-ES-RSF

The time required to complete the PSAS-ES was seen as a potential difficulty in establishing the questionnaire as a systematic screening tool for postpartum anxiety (Costas-Ramon et al. 2023). One of the future research lines proposed after the translation and validation of the PSAS-ES was the validation of a short version of the scale, as had already been done with the 16-item English-language research short-form (Davies et al. 2021b) and a 12-item research short-form for use in global crises (Silverio et al. 2021), and in Persian (Mashayekh-Amiri et al. 2023a, 2023b), and in Jordanian Arabic (Hijazi et al. 2024). Furthermore, the psychometric analysis of the PSAS-ES showed three items with low factor loadings compared to the original English version (Costas-Ramon et al. 2023).

For these reasons, it was deemed important to obtain a short version of the PSAS-ES to provide a psychometrically more robust tool with fewer items, facilitating its completion by women. The present study reports the results of confirmatory factor analyses (CFA) of the PSAS-ES conducted on two independent Spanish samples. In the first sample, the CFA is performed with the 51-item version to obtain a short version of 16 items. In the second sample, a CFA is applied to the short version to check the reliability and validity of this version.

## Method

### Participants

The approval for the study for Sample 1 and Sample 2 were requested and granted by the Research Ethics Committee of the Complutense University of Madrid (Ref: CE_20230112-10_SAL; CE_20240314_26_SOC).

The participants for Sample 1 were 699 women, between 21 to 43 years. (M = 33.39; SD = 3.79). The participants for Sample 2 were 293 women, between 24 to 46 years (M = 34.45; SD = 3.67). Both samples were women between 0 and 16 weeks postpartum (See Table 1).

**Table 1:** Demographic data of women in both samples

Maternal characteristic	Value first sample	Value second sample
**Age (years)**		
Min–Max	21–43	24–46
Mean (*SD*)	33.39 (3.79)	34.45 (3.67)
**Nationality (*n/%*)**		
Spain	679 (97.3)	286 (97.3)
Colombia	4 (0.6)	1 (0.3)
Chile	2 (0.3)	1 (0.3)
Argentina	4 (0.6)	
Dominican Republic	3 (0.4)	
Peru	2 (0.3)	2 (0.7)
Venezuela	3 (0.4)	1 (0.3)
Mexico	1 (0.1)	1 (0.3)
Portugal		2 (0.7)
**Marital status (*n/%*)**		
Single	207 (29.6)	4 (1.4)
Single Cohabiting	111 (15.9)	101 (34.3)
Married	375 (53.6)	185 (62.9)
Separated/divorced	6 (0.9)	4 (1.4)
**Educational attainment (*n/%*)**		
Primary school education	1 (0.2)	4 (1.4)
Secondary studies	135 (19.2)	39 (13.3)
University education	563 (80.5)	251 (85.4)
**Employment situation (*n/%*)**		
Active employment	133 (19.0)	10 (3.4)
Not in paid occupation	59 (8.5)	17 (5.8)
Maternity leave (Maternity Benefit)	507 (72.5)	267 (90.8)
**Birth order (*n/%*)**		
First	530 (75.8)	178 (60.5)
Second	154 (22.0)	103 (35.5)
Third	13 (1.9)	9 (3.1)
Others	2 (0.3)	4 (0.9)
**Multiple birth (*n/%*)**		
Yes	6 (0.9)	5 (1.7)
No	692 (99.1)	289 (98.3)
**Childbirth Modalities (*n/%*)**		
Normal delivery (spontaneous labour)	401 (57.4)	227 (77.2)
Caesarean section	162 (23.2)	67 (22.8)
Instrumental vaginal delivery: vacuum, kiwi, forceps	136 (19.4)	39 (13.3)
**Mode of feeding (*n/%*)**		
Exclusively breastfeeding	505 (72.1)	217 (73.8)
Combination feeding	127 (18.3)	49 (16.7)
Exclusively formula feeding	67 (9.6)	28 (9.5)
**Previous diagnosis of emotional disturbances**		
Yes	90 (13.0)	47 (16.0)
No	554 (79.9)	239 (81.3)

For both samples, women were recruited between 0 and 16 weeks postpartum. They were recruited via word-of-mouth; snowballing; through professional social media platforms related to maternity and the postpartum; and through psychologists, midwives, obstetricians in specialized centers; and mental health forums in the puerperium.

### Materials

The Postpartum Specific Anxiety Scale in Spanish Version (PSAS-ES; Costas-Ramon et al. 2023) is a 51-item self-report questionnaire administered to screen for the frequency of postpartum specific anxieties. The mothers should answer the questionnaire in relation to their emotions, feelings, and experiences of the last seven days. The Scale have four factors: (1) ‘Maternal Competence and Attachment Anxieties’; (2) ‘Infant Safety and Welfare Anxieties’; (3) ‘Practical Infant Care Anxieties’; (4) ‘Psychosocial Adjustment to Motherhood’. The PSAS is rated on a 4-point Likert scale ranging from 1 (never) to 4 (almost always). Higher scores indicate higher levels of postpartum specific anxiety.

The State-Trait Anxiety Inventory (STAI) measures two types of anxiety. It is a self-assessment questionnaire with 40 items and answers are reflected on a 4-point scale: 0-Nothing, 1-Somewhat, 2-Quite a bit, and 3-Very much; where higher scores on the scale correspond to higher levels of anxiety.

The Beck Depression Inventory is a self-report questionnaire which detects and assesses depression. It contains 21 groups of statements where the mothers had to choose the one that best described how they felt in the last two weeks. Each answer has an associated score of 0–3. Higher scores are related to higher levels of depressive symptoms.

### Procedure

The questionnaires were disseminated, completed, and received exclusively on-line through the Google Forms tool. Prior to the main survey, an electronic consent form and information sheet were provided with a tick box to confirm the main points were correct and understood. At the beginning of the questionnaire women accepted the informed consent which detailed the objectives and phases of the study; information about data retention and contact information of the researchers (in case of any queries). Informed consent had to have been provided to continue with the rest of the questionnaires. Participants did not receive any compensation for participating in the study. All procedures performed in this study were in accordance with the ethical standards of the institutional and/or national research committee and with the 1964 Helsinki declaration and its later amendments or comparable ethical standards.

### Method of analysis

With the data from the first sample, we conducted a CFA to extract the 16 items into the 4 original factors that had the highest factor loadings, following the methodology used by the authors of the original research short form scale (Davies et al. 2021b). With the second sample, we conducted another CFA where the structure of the abbreviated version of 16 items was verified.

The CFA was performed with R Studio 4.0.4 from R (R Core Team 2021), using the Lavaan package (Rosseel 2012). The polychoric correlation matrix was used. As parameter estimation method, Diagonally Weighted Least Squares was used, given the number of response categories and the skewness and kurtosis indexes.

To assess the fit of each model individually, the following indicators were considered: the χ2 statistic, the Comparative Fit Index (CFI), the Tucker-Lewis Index (TLI), the Root Mean Square Error of Approximation (RMSEA) and the Root Mean Square Residual (SRMR). For the CFI and TLI indexes, values greater than 0.90 are considered an adequate fit of the model (Shumacher 1996), while for the RMSEA values less than 0.08 are considered a reasonable fit (Browne et al. 1993). To determine the best statistical model, the chi-square test of differences was used.

## Results

### Study 1: confirmatory factor analyses with PSAS-ES 51 items

The factor loadings of the items on each one of the factors were statistically significant and sufficiently high (>. 40). The goodness-of-fit indexes were (χ2 (1218) = 3597.537, p < 0.000, CFI = 0.955, and RMSEA = 0.057, SRMR = 0.071, TLI = 0.953. The correlations between factors are found in Table 2.

**Table 2:** Factor structure of the PSAS-ES

	Factor 1	Factor 2	Factor 3	Factor 4
PSAS_1	0.000	0.000	0.000	0.546
PSAS_2	0.000	0.000	0.000	0.351
**PSAS_3**	0.000	**0.650**	0.000	0.000
PSAS_4	0.544	0.000	0.000	0.000
PSAS_5	0.000	0.000	0.000	0.524
**PSAS_6**	0.000	0.000	0.000	**0.590**
PSAS_7	0.414	0.000	0.000	0.000
PSAS_8	0.000	0.577	0.000	0.000
PSAS_9	0.651	0.000	0.000	0.000
PSAS_10	0.000	0.000	0.000	0.457
PSAS_11	0.597	0.000	0.000	0.000
PSAS_12	0.000	0.000	0.000	0.474
PSAS_13	0.000	0.000	0.650	0.000
PSAS_14	0.000	0.000	0.616	0.000
PSAS_15	0.000	0.625	0.000	0.000
**PSAS_16**	0.000	**0.751**	0.000	0.000
**PSAS_17**	**0.745**	0.000	0.000	0.000
PSAS_18	0.629	0.000	0.000	0.000
PSAS_19	0.000	0.437	0.000	0.000
PSAS_20	0.667	0.000	0.000	0.000
PSAS_21	0.000	0.000	0.000	0.333
PSAS_22	0.000	0.365	0.000	0.000
PSAS_23	0.727	0.000	0.000	0.000
**PSAS_24**	**0.759**	0.000	0.000	0.000
PSAS_25	0.542	0.000	0.000	0.000
PSAS_26	0.000	0.000	0.000	0.411
PSAS_27	0.000	0.486	0.000	0.000
**PSAS_28**	0.000	0.000	**0.721**	0.000
PSAS_29	0.000	0.000	0.000	0.544
PSAS_30	0.000	0.000	0.000	0.560
**PSAS_31**	0.000	**0.789**	0.000	0.000
PSAS_32	0.000	0.000	0.000	0.560
**PSAS_33**	0.000	**0.684**	0.000	0.000
PSAS_34	0.000	0.000	0.601	0.000
PSAS_35	0.000	0.490	0.000	0.000
PSAS_36	0.624	0.000	0.000	0.000
PSAS_37	0.000	0.000	0.000	0.503
PSAS_38	0.592	0.000	0.000	0.000
**PSAS_39**	**0.759**	0.000	0.000	0.000
**PSAS_40**	**0.730**	0.000	0.000	0.000
**PSAS_41**	0.000	0.000	**0.708**	0.000
**PSAS_42**	0.000	0.000	0.000	**0.581**
PSAS_43	0.000	0.000	0.000	0.373
**PSAS_44**	0.000	0.000	0.000	**0.607**
PSAS_45	0.711	0.000	0.000	0.000
PSAS_46	0.000	0.000	0.000	0.526
**PSAS_47**	0.000	0.000	**0.694**	0.000
PSAS_48	0.000	0.000	0.000	0.553
**PSAS_49**	0.000	0.000	**0.699**	0.000
PSAS_50	0.000	0.617	0.000	0.000
**PSAS_51**	0.000	0.000	0.000	**0.667**

The goodness-of-fit indexes were (χ^2^ (1218) = 3335.85, CFI = 0.99, TLI = 0.99 and RMSEA = 0.045, SRMR = 0.071. All factor loadings were highly significant (p < 0.001). Standardised factor loadings are reported in Table 3.

**Table 3:** Standardised factor loadings ordered by strength of association and reliability for each subscale

Item	Factor 1: Maternal competence & attachment anxieties	Factor 2: Infant safety & welfare anxieties	Factor 3: Practical infant care anxieties	Factor 4: Psychosocial adjustment to motherhood
**PSAS 39**	**0.709**			
**PSAS 9**	**0.628**			
**PSAS 24**	**0.618**			
**PSAS 17**	**0.603**			
PSAS 45	0.591			
PSAS 23	0.564			
PSAS 40	0.562			
PSAS 11	0.550			
PSAS 20	0.533			
PSAS 38	0.490			
PSAS 4	0.481			
PSAS 7	0.463			
PSAS 25	0.453			
PSAS 18	0.424			
PSAS 36	0.342			
**PSAS 31**		**0.753**		
**PSAS 16**		**0.671**		
**PSAS 3**		**0.670**		
**PSAS 33**		**0.598**		
PSAS 15		0.573		
PSAS 50		0.560		
PSAS 35		0.540		
PSAS 8		0.523		
PSAS 27		0.428		
PSAS 22		0.406		
PSAS 19		0.307		
**PSAS 28**			**0.637**	
**PSAS 49**			**0.626**	
**PSAS 41**			**0.600**	
**PSAS 13**			**0.595**	
PSAS 34			0.591	
PSAS 47			0.563	
PSAS 14			0.505	
**PSAS 6**				**0.585**
**PSAS 51**				**0.553**
**PSAS 46**				**0.524**
**PSAS 10**				**0.496**
PSAS 30				0.496
PSAS 32				0.484
PSAS 44				0.476
PSAS 29				0.474
PSAS 42				0.473
PSAS 12				0.470
PSAS 1				0.435
PSAS 43				0.417
PSAS 2				0.409
PSAS 48				0.408
PSAS 5				0.396
PSAS 37				0.348
PSAS 21				0.309
PSAS 26				0.235
ω_t_	0.92	0.89	0.89	0.89
α	0.91	0.86	0.83	0.86

The reliability for each subscale was excellent ω_t_ ≥ 0.89 for all subscales. Omega hierarchical was calculated to test the amount of reliable variance in the scale produced by a common factor (g). This was below the acceptable level (ω_h_ = 0.62) suggesting the subscales can be used with confidence, but a total score is less useful. With the results obtained, we retained the 4 items with the highest factor loadings from each of the 4 factors to propose the 16-item version of the PSAS-ES-RSF (Table 4).

**Table 4:** PSAS-ES-RSF

**Factor 1: Maternal competence and attachment anxieties**	
PSAS 39	Me he sentido insegura o incapaz de satisfacer las necesidades básicas de mi bebé *I have felt unconfident or incapable of meeting my baby’s basic care needs*
PSAS 9	He sentido que otras madres cuidan mejor de sus bebés que yo *I have felt that other mothers are coping with their babies better than me*
PSAS 24	Me preocupa que mi bebé note mis ansiedades *I have worried that my baby is picking up on my anxieties*
PSAS 17	Me ha preocupado el vínculo que tengo con mi bebé *I have worried about the bond I have with my baby*
**Factor 2: Infant safety and welfare anxieties**	
PSAS 31	Me ha preocupado hacer daño a mi bebé accidentalmente *I have worried about accidentally harming my baby*
PSAS 16	Me preocupa que alguien o algo, por accidente, haga daño a mi bebé *I have worried about my baby being accidentally harmed by someone or something else*
PSAS 3	Me he preocupado por la salud de mi bebé incluso después de que me tranquilizaran otras personas *I have worried about my baby’s health even after reassurance from others*
PSAS 33	Me preocupa que mi bebé deje de respirar mientras duerme *I have worried that my baby will stop breathing while sleeping*
**Factor 3: Practical infant care anxieties**	
PSAS 28	Me preocupa la cantidad de leche que toma mi bebé *I have worried about my baby’s milk intake*
PSAS 49	Me preocupa el tiempo que duerme mi bebé *I have worried about the length of time that my baby sleeps*
PSAS 41	Me ha preocupado que mi bebé no se desarrolle tan rápido como otros bebés *I have worried that my baby is not developing as quickly as other babies*
PSAS 13	Me he preocupado por la forma en la que alimento a mi bebé *I have worried about the way that I feed my baby*
**Factor 4: Psychosocial adjustment to motherhood**	
PSAS 6	Me ha costado más concentrarme en tareas simples que antes de que mi bebé naciera *I have been less able to concentrate on simple tasks than before my baby was born*
PSAS 51	Me preocupa volver al trabajo *I have worried about returning to work*
PSAS 46	He tenido dificultades para dormir incluso cuando tengo la oportunidad de hacerlo *I have had difficulty sleeping even when I have had the chance to*
PSAS 10	Me he sentido cansada incluso después de un buen descanso *I have felt tired even after a good amount of rest*

### Study 2: confirmatory factor analyses with PSAS-ES-RSF 16 items

**Fig. 1:**
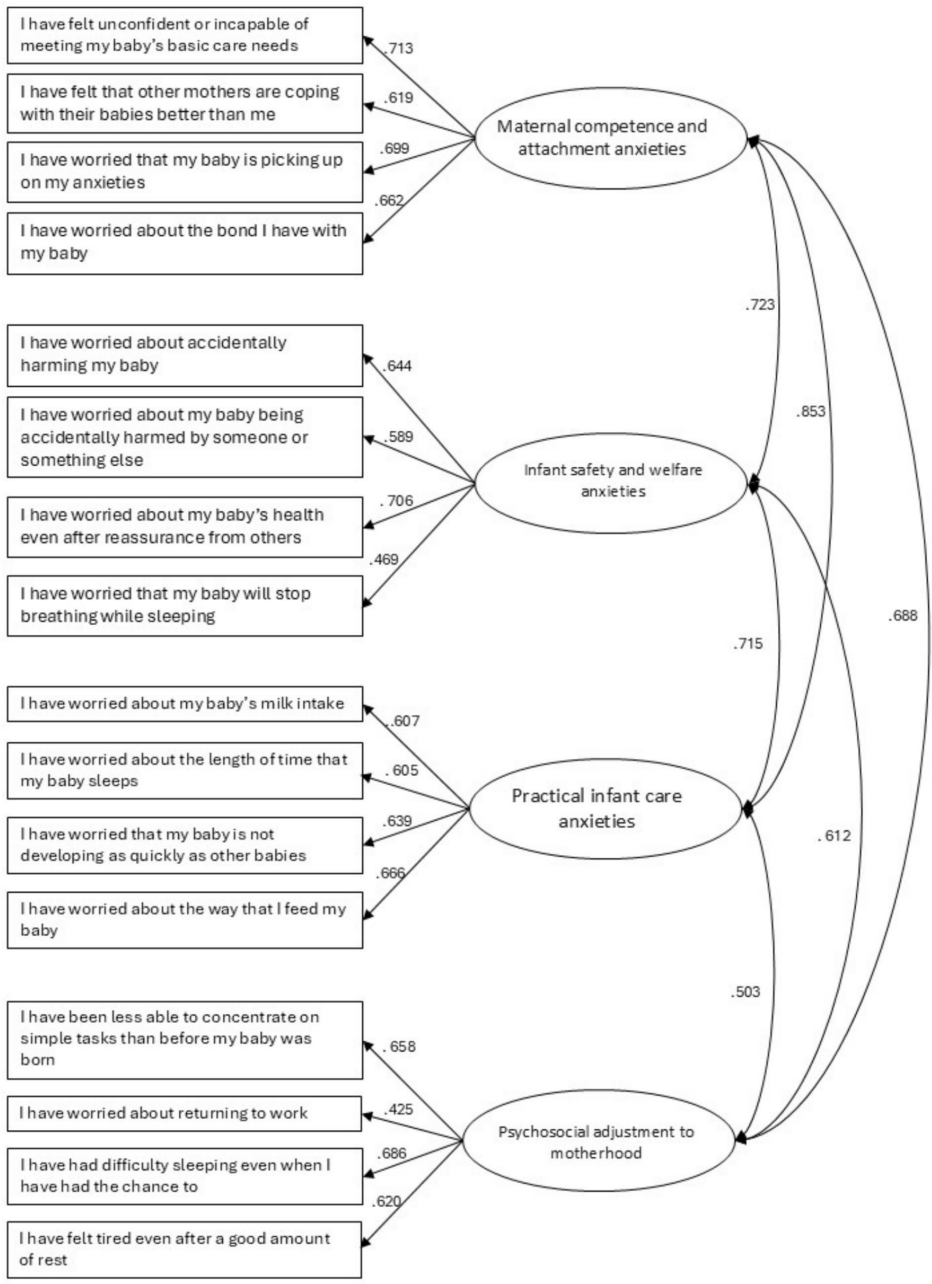
Standardized factor loadings and covariance between factors. Note: The name of the factors are: One: Maternal competence and attachment anxieties; Two: Infant safety and welfare anxieties; Three: Practical infant care anxieties; Four: Psychosocial adjustment to motherhood

With the second sample, the goodness-of-fit indexes of model of four factors were χ2 (98) = 214.082, p < 0.000, CFI = 0.980, and RMSEA = 0.064, SRMR = 0.072, TLI = 0.975. The correlations between factors are found in Fig. 1.

With the second sample, the goodness-of-fit indexes of model of four factors were χ2 (120) = 349.25, p < 0.001, CFI = 0.973, TLI = 0.967 and RMSEA = 0.054, SRMR = 0.054. All factor loadings were highly significant (p < 0.001).

Reliability of the short form was also tested. McDonald omega hierarchical again suggested that subscales should be used rather than the overall scale value (ω_h_ = 0.59). Despite fewer items in the short form, all subscales showed acceptable to good reliability See Table 5.

**Table 5:** Internal reliability of the short form subscales

Factor	ω_t_	α
Maternal competence and attachment anxieties	0.85	0.81
Infant safety and welfare anxieties	0.75	0.73
Practical infant care anxieties	0.81	0.77
Psychosocial adjustment to motherhood	0.80	0.73

The convergent validity of PSAS-ES was analyzed with other theoretically related measures of anxiety (STAI-S, STAI-T) and depression (BDI-II) indicating good convergent validity (see Supplementary Tables 1, 2, and 3).

## Discussion

The items with the highest factor loadings in the Spanish version differ from the English-language research short form, developed from the original PSAS (PSAS-RSF; 16 items). Four items are common to the short versions of both scales: “I worry that my baby will pick up on my anxieties”, “I worry that someone or something will accidentally harm my baby”, “I worry that my baby will stop breathing while asleep”, and “I worry about the amount of milk my baby takes”. The differences in results can be attributed to the cultural context analysis and the risk factors or variables related to postpartum anxiety.

Two items on the PSAS-ES-RSF refer to insecurity and/or inability to meet the baby’s needs and the feeling that other mothers are better able to care for their children. Studies in the Spanish population indicate that lack of self-efficacy in caregiving is related to postpartum anxiety (Feligreras-Alcalá et al. 2020). Specifically, after the birth of their first baby, the psycho-emotional aspects that generate the most concern among women are their low self-perception of being able to care for the baby (Escuriet and Figueroa 2004). Postpartum anxiety affects mother–child interactions, the development of the affective bond between them, and attachment (Field 2017; Davies et al. 2021a; Bonacquisti et al. 2020). This concern about forming an effective bond with their babies is reflected in the item: “I have worried about the bond I have with my baby.”

Work and family are established by many societies as the two most important and prioritized areas of life (Pascual et al. 2016). In this vein, returning to work is a key component of mothers’ anxieties. It should be noted that the current sample are 0–16 weeks postpartum and most of them have not returned to paid work since maternity leave in Spain lasts 16 weeks. It is expected to be an aspect that generates more anxiety compared to the sample of English women (PSAS-RSF; Feild 2017) whose inclusion criterion was the first 12 months postpartum.

According to our results, previous research in Spanish women also finds that breastfeeding is a source of anxiety and concerns after the birth of their children, and feeding, in general, is one of their main worries (Urrutia and Figueroa 2015). One item refers to the concern about how to feed the baby. Even in other cultural contexts, it has been observed that the presence of postpartum anxiety decreases the duration of breastfeeding, reduces self-efficacy, and increases difficulties in breastfeeding (Fallon et al. 2016b).

After the birth of their child, mothers’ main concerns focus on the baby’s health and care, their insecurity in solving problems related to the general care of the child (Pascual et al. 2016; Nunes and Ayala 2007). These findings align with the items which refer to accidentally harming the baby and concern for the child’s health, rest time, and development.

Difficulty sleeping and fatigue are recognized in previous studies as variables related to emotional disorders (Caparros-Gonzalez et al. 2018; Gunst et al. 2021; Parfitt and Ayers 2014).

Physical manifestations related to anxiety: "I have found it harder to concentrate on simple tasks" were also found. The definition of anxiety in the context of the perinatal period includes manifestations such as palpitations, muscle tension, insomnia, and difficulty concentrating (Azúa Morera and Carvajal Barboza 2024). The PSAS doesn’t attempt to measure physiological symptoms because they can be conflated with normal symptoms of motherhood.

### Strengths, limitations, and future directions

The PSAS-ES-RSF, like the original scale PSAS-RSF, allows exploring areas of particular concern for women in the postpartum period, considering the biopsychosocial characteristics of specific vulnerability after the birth of their child. One of the main strengths of the study is the number of women who participated, allowing a robust psychometric analysis.

Furthermore, obtaining a reduced number of items makes it easier for professionals to use and for mothers to complete the questionnaire in a shorter time, potentially increasing the number of responses and being more accessible in a health care setting.

The research presents limitations that must be considered in the design and analysis of future studies. The study participants constitute a homogeneous sample of heterosexual couples with a medium to high sociocultural level (university or higher education). This means that the tool has not been analysed and tested in other population groups. Using the PSAS-ES-RSF in other family models (homosexual couples, single mothers) with different sociocultural levels would help to explore and better understand the needs of other populations.

Future research should continue evaluating cultural differences since our study shows that there are different items in the short versions when comparing the UK and Spanish PSAS. This does not invalidate the instrument (as the full version has been validated for both samples with the same items), but indicates that when reducing the instrument, there are differences in the items which best explain postpartum anxiety in women, depending on the country. In this sense, it is advisable to make cultural adaptations for proposed short versions and not only validate the items already proposed and validated in other countries – something which the PSAS Working Group fully endorses.

It is also proposed to explore the scale’s validity in other time periods to benefit a larger number of women at different times postpartum such as after returning to work.

## Conclusions

The PSAS-ES-RSF 16-item tool demonstrates reliability and construct validity for use in exploring postpartum anxiety during the first 16 weeks postpartum. Its use by healthcare professionals can help distinguish physiological adaptive reactions and experiences from those requiring more complex care plans. This reduced version, compared to the original scale, allows for easier inclusion in systematic screening programs for emotional disorders in the perinatal period.

## Supplementary Information

Below is the link to the electronic supplementary material.

Supplementary file1 (DOCX 35 KB)

## Data Availability

The datasets are not publicly available due to their sensitive nature, however they are available upon reasonable request from the corresponding author.

## Abbreviations

PSAS: Postpartum Specific Anxiety Scale
CFA: Confirmatory Factor Analyses
WLSMV: Diagonally Weighted Least Squares
CFI: Comparative Fit Index
TLI: Tucker-Lewis Index
RMSEA: Root Mean Square Error of Approximation
SRMR: Root Mean Square Residual
STAI-S: State-Trait Anxiety Inventory, State Scale
STAI-T: State-Trait Anxiety Inventory, Trait Scale
BDI-II: The Beck Depression Inventory-II
