# Supplementary material for: Validation of the Spanish-language version of the Postpartum Specific Anxiety Scale Research Short Form: PSAS-ES-RSF
Source: Arch Womens Ment Health. 2026 Mar 30;29(2):57. doi: 10.1007/s00737-026-01695-1 (PMC13035555; doi:10.1007/s00737-026-01695-1)
Supplement: Supplementary file 1 — Supplementary file1 (DOCX 35 KB) [file 737_2026_1695_MOESM1_ESM.docx]

Table 1. Pearson product-moment correlations between the PSAS-ES and STAI-S

|  | PSAS-ES-1 | PSAS-ES-2 | PSAS-ES-3 | PSAS-ES-4 | PSAS-ES-5 | PSAS-ES-6 | PSAS-ES-7 | PSAS-ES-8 | PSAS-ES-9 | PSAS-ES-10 | PSAS-ES-11 | PSAS-ES-12 | PSAS-ES-13 | PSAS-ES-14 | PSAS-ES-15 | PSAS-ES-16 |
| --- | --- | --- | --- | --- | --- | --- | --- | --- | --- | --- | --- | --- | --- | --- | --- | --- |
| STAI1 | -.194* | -.211* | -.213* | -.361* | -.304* | -.115** | -.232* | -.150* | -.362* | -.253* | -.201* | -.166* | -.247* | -.175* | -.210* | -.349* |
| STAI2 | -.236* | -.261* | -.221* | -.532* | -.435* | -.255** | -.305* | -.231* | -.332* | -.339* | -.273* | -.145* | -.262* | -.233* | -.291* | -.300* |
| STAI3 | .210* | .193* | .218* | .359* | .311* | .157* | .256* | .194* | .412* | .302* | .279* | .181* | .295* | .216* | .233* | .366* |
| STAI4 | .262* | .251* | .273* | .388* | .285* | .150* | .300* | .234* | .352* | .312* | .238* | .170* | .338* | .216* | .218* | .341* |
| STAI5 | -.201* | -.269* | -.168* | -.450* | -.344* | -.200* | -.240* | -.193* | -.354* | -.315* | -.233* | -.146* | -.266* | -.188* | -.239* | -.361* |
| STAI6 | .144* | .184* | .198* | .340* | .285* | .120* | .221* | .114* | .376* | .280* | .176* | .141* | .290* | .210* | .152* | .324* |
| STAI7 | .345* | .216* | .415* | .297* | .278* | .204* | .429* | .349* | .331* | .275* | .220* | .249* | .243* | .274* | .242* | .258* |
| STAI8 | -.090 | -.195* | -.045 | -.210* | -.158* | -.106* | -.165* | -.093 | -.234* | -.152* | -.146* | -.087 | -.313* | -.065 | -.142 | -.390* |
| STAI9 | .223* | .242* | .272* | .427* | .386* | .233* | .335* | .231* | .410* | .366* | .249* | .230* | .318* | .254* | .232* | .398* |
| STAI10 | -.210* | -.212* | -.165* | -.379* | -.335* | -.160* | -.249* | -.193* | -.363* | -.284* | -.238* | -.148* | -.299* | -.188* | -.248* | -.349* |
| STAI11 | -.249* | -.183* | -.200* | -.482* | -.477* | -.229* | -.243* | -.216* | -.339* | -.332* | -.282* | -.146* | -.263* | -.264* | -.313* | .337* |
| STAI12 | .158* | .188* | .240* | .353* | .327* | .131* | .245* | .148* | .403* | .308* | .213* | .172* | .299* | .188* | .209* | .389* |
| STAI13 | .127* | .120* | .131* | .281* | .240* | .093 | .090 | .078 | .294* | .218* | .143* | .123* | .242* | .169* | .133* | .234* |
| STAI14 | .116* | .205* | .124* | .218* | .244* | .076 | .153* | .113* | .303* | .219* | .217* | .155* | .266* | .202* | .104* | .314* |
| STAI15 | -.221* | -.230* | -.197* | -.370 | -.318* | -.185* | -.275* | -.160* | -.353* | -.285* | -.263* | -.218* | -.305* | -.194* | -.248* | -.355* |
| STAI16 | -.223* | -.201* | -.172* | -.426* | -.318* | -.238* | -.256* | -.160* | -.361* | -.343* | -.223* | -.131* | -.281* | -.241* | -.274* | -.351* |
| STAI17 | .327* | .254* | .332* | .424* | .428* | .302 | .441* | .302* | .370* | .357* | .322* | .188* | .282* | .303* | .314* | .323* |
| STAI18 | .205* | .246* | .243* | .391* | .343* | .123* | .248* | .127* | .365* | .268* | 3149* | .180* | .354* | .229* | .162* | .349* |
| STAI19 | -.154* | -.189* | -.96 | -.359* | -.339* | -.140* | -.144* | -.080 | -.285* | -.216* | -.193* | -.126* | -.281* | -.150* | -.171*- | -.283* |
| STAI20 | -.145* | -.194* | -.134* | -.373* | -.314* | -.153* | -.166* | -.094 | -.309* | -.267* | -.221* | -.140* | -.268* | -.154* | .158* | -.309* |

*p<.01

Table 2. Pearson product-moment correlations between the PSAS-ES and STAI-T

|  | PSAS-ES-1 | PSAS-ES-2 | PSAS-ES-3 | PSAS-ES-4 | PSAS-ES-5 | PSAS-ES-6 | PSAS-ES-7 | PSAS-ES-8 | PSAS-ES-9 | PSAS-ES-10 | PSAS-ES-11 | PSAS-ES-12 | PSAS-ES-13 | PSAS-ES-14 | PSAS-ES-15 | PSAS-ES-16 |
| --- | --- | --- | --- | --- | --- | --- | --- | --- | --- | --- | --- | --- | --- | --- | --- | --- |
| STAI1 | -,203* | -,221* | -,169* | -,401* | -,344* | -,198* | -,200* | -,111* | -,382* | -,218* | -,170* | -,147* | -,338* | -,213* | -,210* | -,337* |
| STAI2 | ,181* | ,266* | ,174* | ,287* | ,209* | ,133* | ,229* | ,159* | ,290* | ,199* | ,208* | ,180* | ,329* | ,174* | ,179* | ,485* |
| STAI3 | ,203* | ,171* | ,207* | ,389* | ,345* | ,225* | ,255* | ,199* | ,402* | ,205* | ,119* | ,202* | ,328* | ,231* | ,215* | ,373* |
| STAI4 | ,221* | ,187* | ,225* | ,388* | ,410* | ,186* | ,221* | ,136* | ,397* | ,239* | ,131* | ,230* | ,289* | ,234* | ,241* | ,344* |
| STAI5 | ,173* | ,236* | ,190* | ,229* | ,238* | ,131* | ,239* | ,150* | ,271* | ,204* | ,179* | ,159* | ,204* | ,200* | ,128* | ,241* |
| STAI6 | -,093* | -,273* | -,036 | -,235* | -,208* | -,107* | -,156* | -,057 | -,277* | -,206* | -,156* | -,107* | -,355* | -,091 | -,140* | -,444* |
| STAI7 | -,199* | -,150* | -,229* | -,336* | -,270* | -,151* | -,281* | -,184* | -,345* | -,193* | -,135* | -,162* | -,202* | -,193* | -,156* | -,296* |
| STAI8 | ,197* | ,173* | ,240* | ,344* | ,297* | ,141* | ,249* | ,160* | ,404* | ,179* | ,164* | ,204* | ,309* | ,208* | ,156* | ,326* |
| STAI9 | ,275* | ,197* | ,288* | ,285* | ,292* | ,177* | ,309* | ,207* | ,341* | ,172* | ,115* | ,174* | ,263* | ,220* | ,160* | ,251* |
| STAI10 | -,131* | -,170* | -,089 | -,278* | -,301* | -,141* | -,132* | -,057 | -,281* | -,145* | -,151* | -,164* | -,279* | -,131* | -,163* | -,295* |
| STAI11 | ,231* | ,146* | ,304* | ,198* | ,244* | ,170* | ,236* | ,200* | ,256* | ,111* | ,127* | ,110* | ,184* | ,214* | ,163* | ,155* |
| STAI12 | ,247* | ,136* | ,285* | ,386* | ,445* | ,194* | ,276* | ,159* | ,378* | ,207* | ,206* | ,187* | ,227* | ,242* | ,235* | ,287* |
| STAI13 | -,210* | -,187* | -,163* | -,422* | -,397* | -,220* | -,259* | -,184* | -,359* | -,261* | -,165* | -,151* | -,287* | -,238* | -,264* | -,323* |
| STAI14 | ,151* | ,158* | ,123* | ,123* | ,200* | ,088 | ,158* | ,098* | ,178* | ,126* | ,116* | ,144* | ,137* | ,183* | ,077 | ,127* |
| STAI15 | ,245* | ,208* | ,225* | ,367* | ,339* | ,144* | ,227* | ,147* | ,375* | ,207* | ,179* | ,205* | ,333* | ,179* | ,156* | ,358* |
| STAI16 | -,191* | -,209* | -,156* | -,374* | -,332* | -,193* | -,244* | -,149* | -,364* | -,199* | -,165* | -,183* | -,332* | -,223* | -,229* | -,351* |
| STAI17 | ,307* | ,213* | ,320* | ,329* | ,309* | ,221* | ,341* | ,212* | ,373* | ,212* | ,149* | ,207* | ,254* | ,227* | ,211* | ,293* |
| STAI18 | ,208* | ,170* | ,307* | ,171* | ,204* | ,102* | ,251* | ,178* | ,366* | ,203* | ,164* | ,278* | ,273* | ,183* | ,121* | ,285* |
| STAI19 | -,230* | -,170* | -,199* | -,312* | -,299* | -,117* | -,248* | -,155* | -,314* | -,166* | -,147* | -,180* | -,224* | -,201* | -,157* | -,283* |
| STAI20 | ,230* | ,205* | ,277* | ,278* | ,254* | ,135* | ,276* | ,247* | ,405* | ,210* | ,231* | ,252* | ,257* | ,238* | ,156* | ,281* |

*p<.01

Table 3. Pearson product-moment correlations between the PSAS-ES and BDI

|  | **PSAS-ES-1** | **PSAS-ES-2** | **PSAS-ES-3** | **PSAS-ES-4** | **PSAS-ES-5** | **PSAS-ES-6** | **PSAS-ES-7** | **PSAS-ES-8** | **PSAS-ES-9** | **PSAS-ES-10** | **PSAS-ES-11** | **PSAS-ES-12** | **PSAS-ES-13** | **PSAS-ES-14** | **PSAS-ES-15** | **PSAS-ES-16** |
| --- | --- | --- | --- | --- | --- | --- | --- | --- | --- | --- | --- | --- | --- | --- | --- | --- |
| BDI1 | ,162* | ,161* | ,146* | ,390* | ,351* | ,182* | ,199* | ,099 | ,368* | ,190* | ,079 | ,145* | ,334* | ,218* | ,165* | ,281* |
| BDI2 | ,163* | ,162* | ,180* | ,265* | ,294* | ,105* | ,186* | ,117* | ,280* | ,129* | ,195* | ,228* | ,250* | ,164* | ,120* | ,291* |
| BDI3 | ,160* | ,163* | ,146* | ,265* | ,351* | ,133* | ,144* | ,129* | ,355* | ,168* | ,090 | ,151* | ,222* | ,204* | ,139* | ,212* |
| BDI4 | ,183* | ,225* | ,174* | ,317* | ,280* | ,138* | ,215* | ,118* | ,330* | ,213* | ,152* | ,215* | ,320* | ,169* | ,138* | ,332* |
| BDI5 | ,226* | ,203* | ,242* | ,337* | ,376* | ,157* | ,233* | ,150* | ,401* | ,213* | ,110* | ,125* | ,249* | ,227* | ,233* | ,271* |
| BDI6 | ,162** | ,121* | ,128* | ,200* | ,262* | ,144* | ,164* | ,099 | ,211* | ,127* | ,105* | ,106* | ,150* | ,170* | ,130* | ,172* |
| BDI7 | ,180** | ,115* | ,253* | ,297* | ,333* | ,200* | ,248* | ,119* | ,299* | ,120* | ,113* | ,299* | ,213* | ,222* | ,188* | ,262* |
| BDI8 | ,149* | ,115* | ,244* | ,287* | ,353* | ,151* | ,224* | ,148* | ,374* | ,208** | ,146* | ,203* | ,184* | ,199* | ,197* | ,250* |
| BDI9 | ,110** | ,141* | ,095 | ,174* | ,280* | ,026 | ,068 | ,046 | ,231* | ,049 | -,006 | ,054 | ,172* | ,151* | ,061 | ,131* |
| BDI10 | ,148** | ,181* | ,197* | ,306* | ,331* | ,207* | ,219* | ,166* | ,337* | ,165* | ,142* | ,153* | ,276* | ,168* | ,137* | ,329* |
| BDI11 | ,132* | ,225* | ,213* | ,245* | ,239* | ,117* | ,210* | ,153* | ,357* | ,163* | ,190* | ,143* | ,264* | ,175* | ,132* | ,320* |
| BDI12 | ,134** | ,221* | ,208* | ,228* | ,213* | ,041 | ,182* | ,100 | ,290* | ,156* | ,223* | ,097 | ,319* | ,176* | ,117* | ,269* |
| BDI13 | ,160** | ,267* | ,198* | ,284* | ,273* | ,171* | ,219* | ,144* | ,307* | ,193* | ,238* | ,142* | ,268* | ,167* | ,156* | ,305* |
| BDI14 | ,231** | ,166* | ,290* | ,339* | ,446* | ,175* | ,244* | ,205* | ,408* | ,224* | ,147* | ,243* | ,257* | ,248* | ,205* | ,312* |
| BDI15 | ,119** | ,247* | ,160* | ,292* | ,265* | ,155* | ,203* | ,105* | ,281* | ,250* | ,181* | ,115* | ,303* | ,142* | ,110* | ,415* |
| BDI16 | ,085 | ,215* | ,117* | ,197* | ,138* | ,164* | ,198* | ,099 | ,209* | ,229* | ,116* | ,079 | ,343* | ,037 | ,154* | ,318* |
| BDI17 | ,096 | ,186* | ,194* | ,248* | ,317* | ,134* | ,203* | ,144* | ,370* | ,187* | ,143* | ,123* | ,276* | ,163* | ,142* | ,327* |
| BDI18 | ,088 | ,150* | ,108* | ,220* | ,144* | ,018 | ,090 | ,062 | ,188* | ,109* | ,105* | ,083 | ,170* | ,069 | ,113* | ,194* |
| BDI19 | ,156* | ,455* | ,172* | ,222* | ,196* | ,078 | ,212* | ,126* | ,275* | ,168* | ,228* | ,128* | ,281* | ,196* | ,148* | ,355* |
| BDI20 | ,091 | ,288* | ,131* | ,269* | ,178* | ,160* | ,213* | ,151* | ,233* | ,254* | ,190* | ,141* | ,338* | ,117* | ,143* | ,431* |
| BDI21 | ,015 | ,127* | ,128* | ,119* | ,141* | ,026 | ,104* | ,041 | ,103* | ,139* | ,143* | ,015 | ,147* | ,136* | ,115* | ,229* |

*p< .01
